# Capybara Oil Improves Renal Pathophysiology and Inflammation in Obese Mice

**DOI:** 10.3390/nu15132925

**Published:** 2023-06-28

**Authors:** Priscila G. Pereira, Luciana L. Alves, Bianca T. Ciambarella, Kíssila Rabelo, Ana Lúcia R. Nascimento, Alan Cesar N. Moraes, Andressa Bernardi, Fernanda V. Guimarães, Gabriela M. Carvalho, Jemima F. R. da Silva, Jorge J. de Carvalho

**Affiliations:** 1 Ultrastructure and Tissue Biology Laboratory, Institute of Biology, Rio de Janeiro State University, Boulevard Vinte e Oito de Setembro, 87 Fundos, 3° Andar Vila Isabel, Rio de Janeiro 20551-030, RJ, Brazil; 2 Electron Microscopy Laboratory of Biology Institute, University of Federal Fluminense, Rio de Janeiro 21040-900, RJ, Brazil; 3 Inflammation Laboratory, Fiocruz, Rio de Janeiro 21040-900, RJ, Brazil

**Keywords:** obesity, kidney injury, capybara oil, omega-3, inflammation, fibrosis

## Abstract

Obesity is an inflammatory disease associated with secondary diseases such as kidney disease, which can cause lipotoxicity, inflammation and loss of organ function. Polyunsaturated fatty acids act in the production of lipid mediators and have anti-inflammatory characteristics. In this work, the objective was to evaluate renal histopathology in obese mice and the effects of treatment with capybara oil (CO) (5000 mg/kg/day for 4 weeks). Parameters such as body mass, lipid profile, systolic blood pressure, urinary creatinine and protein excretion, structure and ultrastructure of the renal cortex, fibrosis, tissue inflammation and oxidative stress were analyzed. CO treatment in obese mice showed improvement in the lipid profile and reduction in systolic blood pressure levels, in addition to beneficial remodeling of the renal cortex. Our data demonstrated that CO decreased inflammation, oxidative stress and renal fibrosis, as evidenced by quantifying the expression of TNF-α, IL-10, CAT, SOD, α-SMA and TGF-β. Although treatment with CO did not show improvement in renal function, ultrastructural analysis showed that the treatment was effective in restoring podocytes and pedicels, with restructuring of the glomerular filtration barrier. These results demonstrate, for the first time, that treatment with CO is effective in reducing kidney damage, being considered a promising treatment for obesity.

## 1. Introduction

Obesity is a complex chronic disease characterized by excess body fat/adiposity harmful to health, as it increases the risk of long-term medical complications and decreases life expectancy [1]. Data from the World Health Organization [2] indicates that about 600 million people worldwide, aged 18 or over, are obese. In 2017, obesity and overweight were the cause of more than 4 million deaths worldwide [3]. It is believed that the main factor capable of leading to obesity is caloric and energy consumption. It is now recognized that the development of obesity is a complex interaction of biological and psychosocial factors [4,5]. Its incidence can lead to the development of secondary diseases such as metabolic syndrome and chronic kidney disease [6,7].

Chronic kidney disease generated by obesity occurs mainly as a consequence of the imbalance between lipolysis and lipogenesis, generating an accumulation of lipids in the kidney. Excess lipids can cause lipotoxicity, triggering an inflammatory process and loss of organ function, which can lead to changes in urinary creatine and protein levels, for example [8]. In addition, the metabolic demand required with increasing body mass leads to the process of hyperfiltration, increasing intraglomerular pressure and, consequently, the risk of kidney damage, especially in the tubules [9,10].

The exact mechanism by which obesity leads to chronic kidney disease has not been fully elucidated. Data in the literature points to reduced production of adiponectin and excessive production of leptin by adipocytes as triggers for renal inflammatory disease. There is intense oxidative stress associated with abnormal lipid metabolism and overactivation of the renin–angiotensin–aldosterone system, in an attempt to balance kidney function altered by inflammation [11,12]. Mice fed a high-fat diet showed albuminuria and renal pathophysiological changes, such as lipid accumulation, increased deposition of type IV collagen in the glomeruli and macrophage infiltration in the renal medulla [13].

Polyunsaturated fatty acids (PUFAs), such as omega-3 and omega-6, are essential for the individual and act as precursors for the production of lipid mediators. These mediators, such as resolvins and lipoxins, have anti-inflammatory characteristics, such as inhibiting the production of pro-inflammatory cytokines, activating transcription factors, recruiting macrophages [14], in addition to modulating the release of reactive oxygen and nitric oxide species [15]. In a previous study with obese mice, it was found that lipoxin is able to attenuate the inflammatory process, reducing albuminuria, free radical production and tubulointerstitial collagen deposition [16].

In patients with chronic kidney disease, a diet rich in omega-3 led to increased production of anti-inflammatory mediators. As humans are not able to synthesize these compounds, a diet rich in PUFAs for these patients becomes essential. The sources of PUFAs are animal and vegetable; however, their extraction process is complex and generates a small amount of final product [17].

In this context, the use of capybara oil (CO) as an alternative source of PUFAs is promising, since it can be extracted in large concentrations. According to Fukushima et al. [18], the oil is extracted from capybara fat and contains 17.9% alpha-linolenic fatty acid and 19.6% linoleic fatty acid, which correspond to omega-3 and omega-6 PUFAs, respectively. In addition, the oil is also made up of oleic (35.6 to 39.8%) and palmitic (20.7 to 24.3%) PUFAs [19]. There are still few studies using CO, but data in the literature shows that it is able to accelerate the healing process in skin wounds in mice [20] and improve steatosis, inflammation and mitochondrial activity liver [21].

Based on this information, we aim to evaluate the renal pathophysiological changes in C57Bl/6 mice with chronic kidney disease, caused by obesity, and the possible beneficial effects of CO in the treatment of this disease.

## 2. Materials and Methods

### 2.1. Obtaining Capybara Oil

Subcutaneous capybara fat was donated by “Santa Luzia Farm” from Goias State, Brazil. Animals were bred in captivity as authorized by “Brazilian Institute of Environment and Renewable Natural Resources”. The fat was extracted using hydrothermal processing in a water bath for liquid oil for oral treatment of mice [21].

### 2.2. Experimental Groups and Diets

All experimental procedures involving animals were approved by the Committee for Ethics in Animal Experimentation of the State University of Rio de Janeiro (n° 031/2018). Twenty four C57BL/6 male mice (3 months old) were maintained in a temperature controlled environment (21 ± 2 °C) and controlled undergoing a reversed light cycle (12 light/dark), in accordance with the Committee’s guidelines. The diets were manufactured by Pragsoluções (Jau, São Paulo, Brazil) following the American Institute of Nutrition recommendations for adult rodents, AIN93M, as detailed in Table 1 [22]. The animals consumed 4 g daily diets for 18 weeks (free access to water) (*n* = 12/group): control diet (CD) and high-fat diet (HFD). The CD contained 10% of total energy from lipids, while the HFD had an addition of lipids (from lard), totaling 60% of the total energy from lipids.

### 2.3. Treatment with Capybara Oil and Experimental Groups

In the last 4 weeks (from 15th to 18th weeks), mice were subdivided into four groups according to treatment administration (*n* = 6/group): CD, CD treated with capybara oil (CD + CO), HFD and HFD treated with capybara oil (HFD + CO). The capybara oil was administered daily via orogastric gavage in the CD + CO and HFD + CO groups at a dose of 5000 mg/Kg for four weeks. CD and HFD groups received water by oral gavage to also be handled.

### 2.4. Body Mass and Systolic Blood Pressure

Weekly body mass (BM) was measured in precision balance to calculate daily capybara oil intake and to evaluate body mass gain.

During the first 14 weeks (pre-treatment period), the systolic blood pressure (SBP) of mice was measured monthly by the noninvasive caudal artery plethysmography method (Kent Scientific, EUA) in all experimental groups with conscious animals. During the treatment period (from 14th to 18th weeks), SBP was measured biweekly to assess the effect of capybara oil on blood pressure evolution. The mean of three measurements of each animal was used. Before the experimental period, the SBP was measured in animals with the objective of acclimatizing them and attenuating possible future changes in pressure due to stress.

### 2.5. Assessment of Renal Function

One day before euthanasia, the groups of animals were placed in metabolic cages for 24 h urine collection (*n* = 3/cage). Urinary creatinine and protein concentrations were determined accordingly with the manufacturer. All commercial kits used were purchased from Bioclin (Bioclin, Belo Horizonte, Minas Gerais, Brazil).

### 2.6. Euthanasia

On the day of euthanasia, 18 weeks after the administration diets, the animals were anesthetized with intraperitoneal injection of Pentobarbital (40 mg/Kg) and the blood was removed by cardiac puncture. Plasma was obtained by centrifugation (1200× *g*, 15 min) and stored at −80 °C. Then, the left kidneys were collected and frozen at −80 °C for oxidative stress assays. In addition, the right kidneys were collected and fixed in 4% paraformaldehyde for optical analysis or 2.5% glutaraldehyde for electron microscopy analysis.

### 2.7. Biochemical Measurement

Total cholesterol (TC), low-density lipoprotein (LDL), high-density lipoprotein (HDL), very-low-density lipoprotein (VLDL) and triglycerides (TGs) in plasma were measured by a colorimetric enzymatic assay according to manufacturer’s recommendations (Bioclin, Brazil).

### 2.8. Morphological Analysis

The kidneys were collected, fixed with 4% buffered paraformaldehyde. For histological processing, kidney samples were dehydrated in an increasing series of 70% (24 h), 90% (1 h) and 100% (1 h) ethanol and diaphanized in xylol 2× (15 min each) to then be soaked in paraffin 2× (30 min each). After embedding in paraffin, blocks containing kidney samples were sectioned into 5 μm-thick slices. The slices were stained with Hematoxylin–Eosin (HE) for histopathological analysis, with Periodic Acid Schiff (PAS) for glomerular basement membrane analysis or Picro Sirius Red (PSR) for analysis of renal fibrosis, and then observed under a light microscope equipped with a CCD camera (Olympus BX53 with camera Olympus DP72, Nagano, Chubu, Japan). The Image-Pro Plus 7.0 program (Media Cybernetics, Silver Springs, Maryland, EUA) was used to obtain the HE, PSR and PAS images with a magnification of 20×, 40× and 40×, respectively.

### 2.9. Immunohistochemistry

The slides with the sections were diaphanized and hydrated. Antigen retrieval was performed by citrate buffer at pH 6.0 incubation for 30 min at 60 °C. Endogenous peroxidase activity was blocked using 0.3% hydrogen peroxide (H_2_O_2_) and non-specific binding of the polyclonal antibodies was blocked by incubation 5% (*w*/*v*) BSA. Subsequently, sections were incubated with antibodies, and these reactions were amplified using a biotin–streptavidin system (Dako, Santa Clara, CA, USA). Immunoreactive products were visualized using diaminobenzidine (DAB) reagent (Dako) and counter-stained with hematoxylin. The slides were dehydrated and mounted with entellan mounting medium. Control sections were obtained by primary antibody omission. We used anti-TNF-α (sc-52746), anti-TGF-β (sc-130348), IL-10 (sc-8438) and anti-α-SMA (sc-53142) (dilution 1:200, Santa Cruz Biotechnology, Dallas, Texas, EUA) antibodies. Then, ten random fields were obtained from each slide and observed under a light microscope. The expression of all markings was quantified using the Image-Pro Plus 7.0 program at a magnification of ×20.

### 2.10. Oxidative Stress Assay

Kidney samples were homogenized using a tissue homogenizer in 500 μL of potassium phosphate + EDTA (KPE) buffer (pH 7.5), and then were centrifuged at 600× *g* for 10 min at 4 °C, the supernatant collected, and the pellet discarded. At the end, the samples were stored at −20 °C until the moment of the analyses, as performed by Kennedy-Feitosa et al. (2016) [23]. Superoxide dismutase (SOD) activity was assayed by monitoring adrenaline (Sigma-Aldrich, St. Louis, MO, USA) inhibition auto-oxidation. The activity is determined by inhibition of the epinephrine self-peroxidation product during auto-oxidation. For the measurement of SOD activity, glycine buffer (pH 10.2) was used and the tissue samples were arranged in three different volumes (1 μL, 2 μL and 3 μL). Then, 193 μL of glycine buffer, 2 μL of catalase and 4 μL of epinephrine were added and the reading was done immediately in a spectrophotometer (SpectraMax M5—Molecular Devices) at 480 nm. As a control, a blank was made where the free oxidation of epinephrine was evaluated without the samples. Catalase (CAT) activity was measured by decrease of H_2_O_2_ (Sigma-Aldrich, St. Louis, MO, USA) rate, and concentrations was monitored. The activity of the catalase enzyme was determined by the rate of decay of hydrogen peroxide (H_2_O_2_) at known concentrations from the first minute of the reaction (1 min). For this assay, “MIX” was prepared, containing 25 mL of distilled water and 40 μL of hydrogen peroxide. Subsequently, 1 μL of sample was added to 99 μL of the MIX. The samples were read in a spectrophotometer (SpectraMax M5—Molecular Devices) at 240 nm absorbance using an UV plate.

### 2.11. Electron Microscopic Study

Kidneys were collected, cut into small tissue blocks (1 mm^3^), and fixed in 2.5% glutaraldehyde at 4 °C. For transmission electron microscope, the fragments were washed in 0.1 M sodium cacodylate buffer, after, postfixaded with 2% osmium tetroxide, tissues were dehydrated in increasing series of acetone (30, 50, 70, 90 and 100%), 15 min each step, and embedded in epoxy resin for three days at 60 °C. Ultrathin sections were contrasted with uranyl acetate and lead citrate. Sections were examined with a JEM1011 electron microscopy (JEOL, Akishima, Tokyo, Japan).

For scanning electron microscopy, the fragments were washed in 0.1 M sodium cacodylate buffer, postfixed with 1% osmium tetroxide diluted in 0.1 M sodium cacodylate buffer. After further washing, the material was dehydrated in an increasing series of ethanol (30, 50, 70, 90% and 2× absolute), 30 min each step. The material was taken to the critical point device for the replacement of ethanol by CO_2_, later fixed in stubs with carbon tape and metallized with gold. After metallization, the material was analyzed using a scanning electron microscope JEOL-JSM-6390-LV (JEOL, Akishima, Tokyo, Japan).

### 2.12. Statistical Analysis

Data were expressed as mean ± standard deviation. Statistical analyzes were performed by comparing the groups, and differences between them were tested. Data were compared by Student’s t-test (pre-treatment period) or two-way ANOVA (post-treatment period) followed by Holm Sidak’s post-test. The significance level used was 5%. Graphpad Prism version 6.0 software (GraphPad Software, San Diego, CA, USA) was used to perform statistical analysis and graphing.

## 3. Results

### 3.1. Physiological Analysis

**Figure 1:**
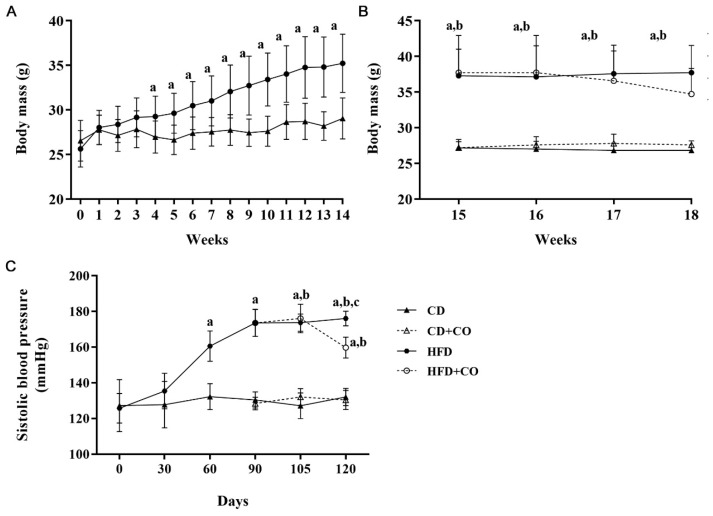
Evolution of body mass and systolic blood pressure. (**A**,**B**) Evolution of BM through 18 weeks in grams (g). (**A**) Body mass in pretreatment period. From the 4th week, the HFD animals had a significant increase in BM when compared to the CD. (**B**) Body mass in post-treatment period. The CD and CD + CO groups showed constant and significantly lower body mass than the HFD and HFD + CO groups. The HFD + CO group showed a non-significant reduction in body mass compared to the HFD group. (**C**) Systolic blood pressure measured in pre- and post-treatment period in millimeters of mercury (mmHg). The HFD group showed a significant increase in SBP compared to the CD group in the pre-treatment period. In the post-treatment period, the HFD and HFD + CO groups showed a significant increase in relation to the CD and CD + CO groups. The HFD + CO group showed a significant reduction in levels in the last week of treatment when compared to the HFD group. (a) represents *p* < 0.05 compared to the CD group, (b) represents *p* < 0.05 compared to the CD + CO group and (c) *p* < 0.05 compared to the HFD + CO group. *n* = 6 for all experimental groups.

The BM of the mice was verified weekly throughout the experimental period (week 0 to week 18). One day before the diets administration, the BM was measured (week 0) (Figure 1A,B).

In the pre-treatment period (week 0 to week 14) (Figure 1A), the BM of the CD group animals remained constant. The HFD group animals increased significantly over the weeks compared to the CD group mice from the 4th week of the experiment.

In the post-treatment period (week 15 to week 18) (Figure 1B), the BM of the CD and CD + CO mice remained constant. The BM of the HFD and HFD + CO groups animals remained significantly higher when compared to the CD and CD + CO groups. The HFD + CO group animals showed a reduction in BM at week 18; however, this reduction was not significant in relation to the HFD group. Thus, the treatment with CO is not able to significantly change the body mass of C57Bl/6 mice.

The SBP of the mice was measured monthly throughout the experimental period from week zero. In the post-treatment period, SBP was measured biweekly in order to monitor the effect of treatment on the evolution of hypertension (Figure 1C). In the pre-treatment period, the SBP of the animals of the HFD group increased progressively and significantly (173.5 ± 7.6 mmHg) compared to the animals of the CD group, which remained constant (130.3 ± 4.5 mmHg). In the post-treatment period, the SBP of the animals in the groups fed the control diet (CD and CD + CO) remained constant during all the weeks of the experiment (133 ± 4.85 mmH and 130.9 ± 4.87 mmHg, respectively) and showed no significant differences between them. On the other hand, the SBP of animals in groups fed a high-fat diet (HFD and HFD + CO) showed a significant increase compared to animals fed a control diet. However, the animals in the HFD + CO group showed a significant reduction in SBP levels in the last week of treatment when compared to the HFD group (159.66 ± 5.85 mmH and 176 ± 4.1 mmH, respectively).

### 3.2. Plasma Biochemical Analysis

Plasma levels of total cholesterol, HDL, LDL, VLDL and triglycerides were evaluated in animals from all experimental groups (Table 2). The HFD group showed a significant increase in the levels of total cholesterol, LDL, VLDL and triglycerides when compared to the groups fed a control diet (CD and CD + CO). In the HFD + CO treated group, a significant decrease in total, LDL and VLDL cholesterol levels was observed compared to the HFD group. In the analysis of triglycerides, the HFD + CO group showed a decrease in levels compared to the HFD group, but this decrease was not significant. In relation to HDL, considered good cholesterol, the HFD + CO group showed a significant increase in relation to the CD and HFD groups.

### 3.3. Assessment of Renal Function

**Figure 2:**
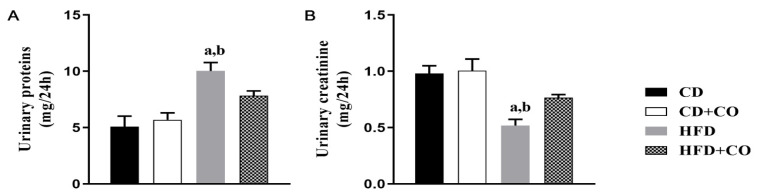
Biochemical parameters in urine. (**A**) Urinary protein levels (mg/24 h). Levels of urinary protein where significant increase in the HFD group compared to the CD and CD + CO groups. HFD + CO group showed a reduction in protein excretion, but not significant in relation to the HFD group levels. (**B**) Creatinine levels (mg/dL). CD and CD + CO were significantly higher when compared to the HFD group. HFD + CO group showed an increase in creatinine excretion compared to the HFD group, but was not significant. (a) *p* < 0.05 compared to the CD group and (b) *p* < 0.05 compared to the CD + CO group. *n* = 6 for all experimental groups.

Urinary levels of creatinine and total proteins were evaluated in the animals of the four experimental groups (Figure 2). Urinary protein levels showed a significant increase in the HFD group compared to the CD and CD + CO groups. The animals in the HFD + CO group showed a reduction in protein excretion, but this decrease was not significant in relation to the HFD group (Figure 2A). The results regarding urinary creatinine levels showed a significant decrease in their excretion in the HFD group when compared to the CD and CD + CO groups. The animals in the HFD + CO group showed an increase in creatinine excretion compared to the HFD group, but this increase was not significant (Figure 2B).

### 3.4. Histopathological Evaluation

**Figure 3:**
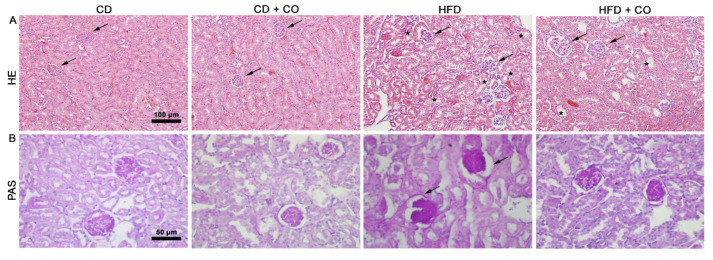
Renal histopathology. (**A**) Coloration in Hematoxylin-Eosin (HE). The CD and CD + CO groups showed preservation of Bowman’s capsule, glomeruli (arrows) and proximal and distal convoluted tubules, unlike the HFD group, which showed areas with possible lipid vesicles (asterisks). The HFD + CO group showed beneficial remodeling of the renal cortex when compared to the HFD group. (**B**) Coloration in Periodic Acid Schiff (PAS). The HFD group showed an intensely stained basement membrane, mainly involving the parietal leaflet of Bowman’s capsule (arrows) and in the glomerular capillaries. The HFD + CO group showed preservation of the basement membrane of the capsule, glomeruli, and proximal and distal convoluted tubules. Calibration bar: 100 µm (HE) and 50 µm (PAS).

Histopathological changes were analyzed by HE staining. The groups CD and CD + CO presented Bowman’s capsule, glomeruli (arrows) and proximal and distal convoluted tubules preserved. However, the HFD group showed a loss in the structure of renal corpuscles and tubules, in addition to possible areas with lipid vesicles (asterisks). In the HFD + CO group, a decrease in areas with lipid vesicles and a beneficial remodeling of the renal cortex was observed when compared to the HFD group, mainly in the proximal and distal convoluted tubules and corpuscles (Figure 3A).

PAS staining was done to highlight basement membranes of glomerular capillary loops and tubular epithelial. The HFD group presented an intensely stained basement membrane, mainly involving the parietal leaflet of Bowman’s capsule (arrows) and in the glomerular capillaries, compared to the CD and CD + CO groups. However, the HFD + CO group showed preservation of the basement membrane of the capsule, of the glomeruli and of the proximal and distal convoluted tubules, presenting a coloration compared to the groups fed a control diet (Figure 3B).

### 3.5. Evaluation of Renal Ultrastructure

**Figure 4:**
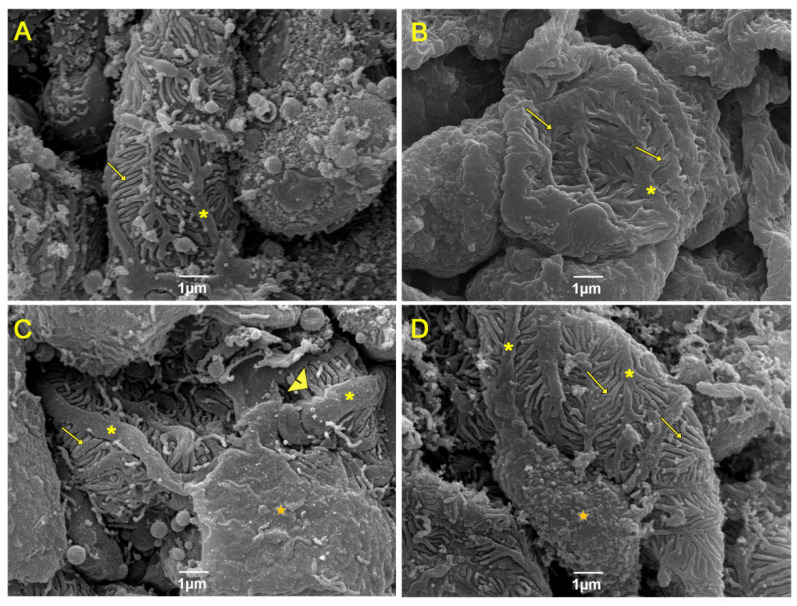
Analysis of renal ultrastructure by scanning electron microscopy. (**A**) Control group; (**B**) Control + CO group; (**C**) HF group; and (**D**) HF + CO group. The CD and CD + CO groups showed preservation of the renal cortex and its cells. The HFD group showed alterations in the cortex cells, showing a more dilated filtration slit and pedicels with irregular contours. The HFD + CO group showed beneficial remodeling of primary and secondary processes compared to the HFD group. Podocytes (*), pedicels (arrows), cell body (star) and glomerular filtration cleft dilatation (arrowhead) are indicated. Calibration bar: 1 µm.

In the analysis of renal ultrastructure by scanning electron microscopy (Figure 4), the control groups (Figure 4A,B) presented intact podocytes (asterisk) consisting of primary and secondary processes (pedicelles) (arrow). In addition, the cell body (star) and the filtration slits were preserved between the regular interdigitations of the pedicels. However, the HF group (Figure 4C) had a more dilated filtration slit (arrowhead) and pedicels with irregular contours, and many did not present regular interdigitations, as well as thickening of the primary processes (asterisk). The HF group treated with CO (Figure 4D) showed beneficial remodeling of the primary and secondary processes, with regular pedicel interdigitations. Additionally, the primary prolongations were thinner and elongated compared to the HF group.

**Figure 5:**
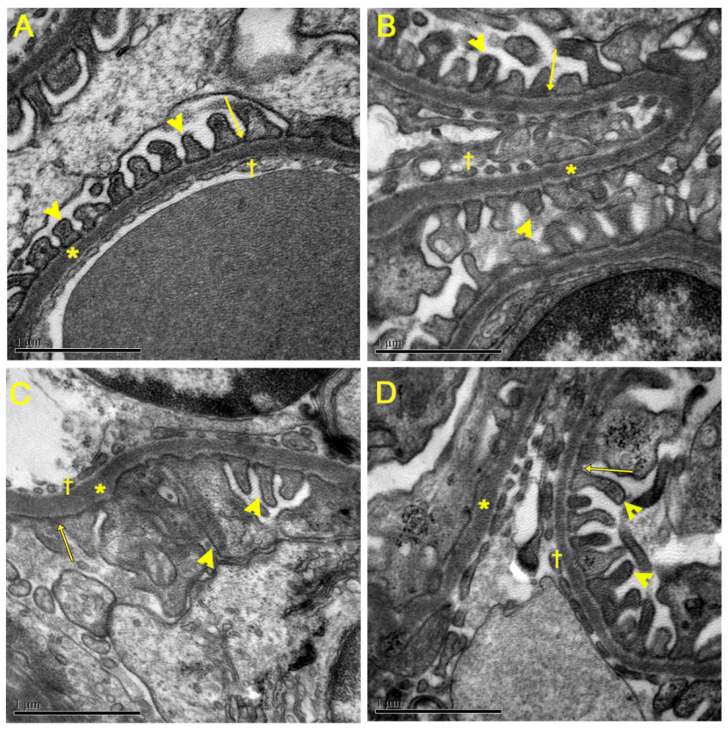
Analysis of renal ultrastructure by transmission electron microscopy. (**A**) Control group; (**B**) Control + CO group; (**C**) HF group; and (**D**) HF + CO group. The CD and CD + CO groups showed preservation of the pedicels and filtration slits with evident membranes. The HFD group presented alterations in the renal ultrastructure, with thickening of the glomerular basement membrane, irregularity in the pedicels and discontinuity of the pores of the glomerular capillaries. The HFD + CO group showed beneficial remodeling of pedicel morphology with preserved filtration slits and membranes. The filtration slit (arrows), pedicels (arrowheads), glomerular basal membrane (*) and fenestrated glomerular capillary pores (†) are indicated. Calibration bar: 1 µm.

In the analysis of renal ultrastructure by transmission electron microscopy (Figure 5), the animals in the control groups (Figure 5A,B) presented numerous pedicels (arrowhead) with regular thicknesses forming the filtration slits (arrow) with evident membranes. Furthermore, it was observed that the glomerular basal membrane (asterisk) is intact and the pores of the glomerular capillaries pores (cross) were continuous. However, the HF group (Figure 5C) presented alterations in the renal ultrastructure, such as thickening of the glomerular basal membrane, variable and irregular sizes of the pedicels (arrowhead), where pedicels with different thicknesses are observed. Additionally, there is discontinuity of the pores of the fenestrated glomerular capillaries. The HF group treated with CO (Figure 5D) showed beneficial remodeling of pedicel morphology (arrowhead) with filtration slits and preserved membranes. In addition, integrity of the glomerular basal membrane, as well as continuity of glomerular capillary pores was observed when compared to the HF group.

### 3.6. Renal Inflammatory Response Evaluation by Immunohistochemistry

**Figure 6:**
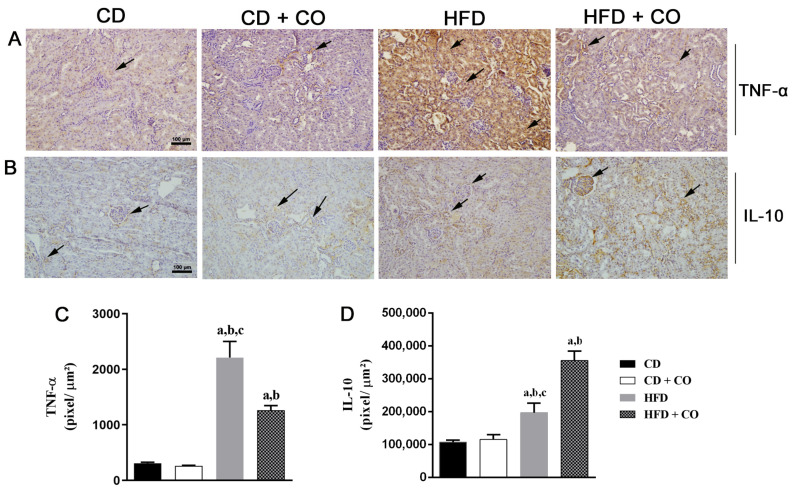
Immunostaining and quantification of anti-TNF-α and anti-IL-10 antibodies in the renal cortex of experimental groups. (**A**) Photomicrographs of TNF-α-specific immunostaining. (**B**) Photomicrographs of specific immunostaining for IL-10. (**C**) Immunostaining quantification (pixel/µm^2^) for anti-TNF-α antibody. TNF-α immunostaining was confirmed by quantification, where levels were significantly higher in the HFD group compared to others. The HFD + CO group showed decreased levels compared to the HFD group. (**D**) Quantification of immunostaining (pixel/µm^2^) for anti-IL-10 antibody. IL-10 immunostaining was confirmed by quantification, where HFD levels were significantly higher when compared to CD and CD + CO. The HFD + CO group showed an increase. Arrows indicate positive immunostaining. (a–c) represent *p* < 0.05 in relation to the CD, CD + CO and HFD + CO groups, respectively. *n* = 6 for all experimental groups. Calibration bar: 100 µm.

In the specific immunostaining for TNF-α and IL-10 (Figure 6A and Figure 6B, respectively), the groups fed a control diet (CD and CD + CO) showed a small expression of these cytokines. In contrast, the HFD group showed many areas of intense TNF-α staining (arrows) in the proximal and distal convoluted tubules and glomeruli and few areas of IL-10 staining. However, the animals in the HFD + CO group showed less frequent staining of TNF-α and high expression of IL-10 when compared to the HFD group. The quantification of TNF-α and IL-10 are represented in Figure 6C and Figure 6D, respectively. The quantitative analysis corroborates the findings described above, with a significant increase in TNF-α levels in the HFD group and IL-10 in the HFD + CO group when compared to the other groups. The groups fed with a control diet (CD and CD + CO) did not show any significant difference between them.

### 3.7. Analysis of the Activity of Enzymes Related to Oxidative Stress

**Figure 7:**
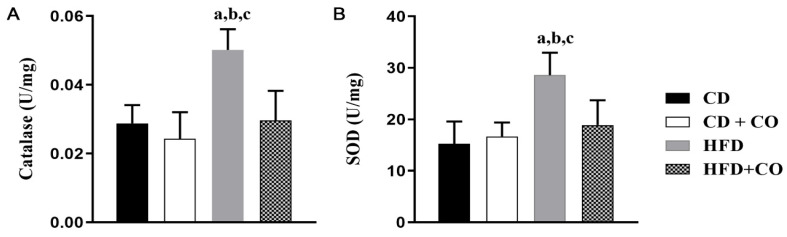
Evaluation of the activity of enzymes related to oxidative stress (U/mg). (**A**) Quantification of the activity of the antioxidant enzyme CAT. (**B**) Quantification of SOD antioxidant enzyme activity. The CD and CD + CO groups showed no significant difference in CAT and SOD levels between them. The HFD group showed a significant increase in the activity of these enzymes compared to the other groups. The HFD + CO group showed a significant decrease compared to the HFD group. (a–c) represent *p* < 0.05 in relation to the CD, CD + CO and HFD + CO groups, respectively. *n* = 6 for all experimental groups.

In the analysis of the enzymatic activity of central proteins associated with oxidative stress (Figure 7), the groups fed a control diet (CD and CD + CO) did not show any significant difference between them. The HFD group showed a significant increase in CAT (Figure 7A) and SOD (Figure 7B) activity compared to the other groups. Regarding the HFD+CO group, there was a significant decrease in both SOD and CAT enzyme activity compared to the HFD group.

### 3.8. Renal Fibrosis Analysis

**Figure 8:**
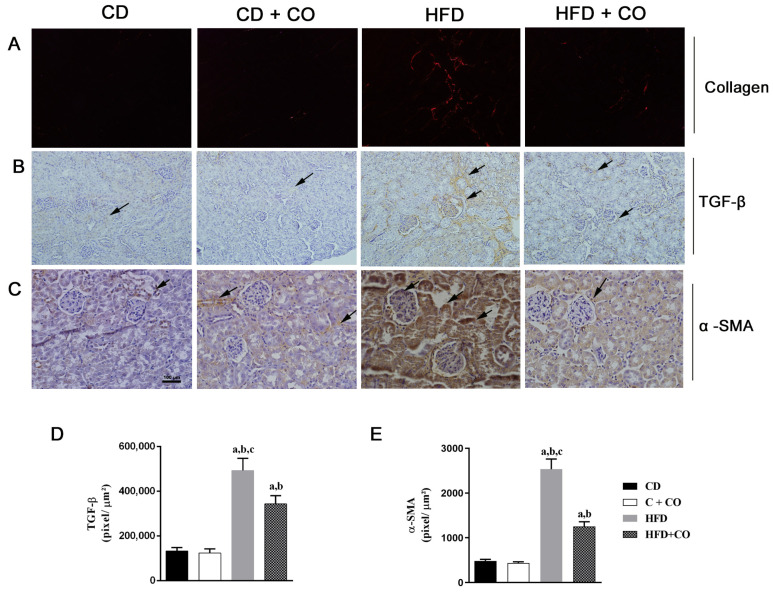
Renal fibrosis analysis in all groups. (**A**) Coloration in Picro Sirius Red (PSR), (**B**) immunostaining of anti-TGF-β, (**C**) immunostaining of anti-α-SMA, (**D**) quantification of immunostaining (pixel/µm^2^) for anti-TGF-β and (**E**) quantification of immunostaining (pixel/µm^2^) for anti-α-SMA. Arrows indicate positive immunostaining mainly in tubules. An increase in collagen deposition was observed in the HFD group compared to the other groups. In an immunohistopathological study, a significant increase in TGF-β and anti-α-SMA levels was observed in the HFD group when compared to the CD and CD + CO groups. In the HFD + CO group, a significant decrease in this microfilament was observed when compared to the HFD group. (a–c) represent *p* < 0.05 in relation to the CD, CD + CO and HFD + CO groups, respectively. *n* = 6 for all experimental groups. Calibration bar: 100 µm (anti-TGF-β) and 50 µm (anti-α-SMA and PSR).

The deposition of collagen fibers was analyzed by PSR staining (Figure 8A), where an increase in collagen deposition can be observed in the HFD group compared to the other groups. In an immunohistopathological study, positive immunostaining of the anti-TGF-β and anti-α-SMA antibody was observed in the renal cortex in all experimental groups (arrows) (Figure 8B and Figure 8C, respectively). The HF group showed intense labeling mainly in the tubules, when compared to the groups fed with a control diet (C and C + CO). In contrast, a decrease in marking areas was observed in the hyperlipidic group treated with CO when compared to the HF group. Through the quantification of the anti-TGF-β and anti-α-SMA antibody, a significant increase in the number of labeled cells was observed in the HF group in relation to the C and C + CO groups, as well as a significant decrease of this microfilament in the HF + CO group when compared to the HF group, as shown in Figure 8D and Figure 8E, respectively.

## 4. Discussion

The prolonged HF diet in C57Bl/6 mice is an established model of diet that induces obesity [24]. Feeding with high lipid content (60% of total energy) to mice is known to induce several systemic alterations, such as obesity, hyperglycemia, abnormal lipid profile, renal alterations, including albuminuria and glomerular lesions [13], in addition to increased SBP [25]. Functional changes are intensified by the degree and time of onset of obesity and by the pro-inflammatory and pro-fibrotic environment typical of obesity. The final result is morpho-structural glomerular alterations, which can manifest in the form of obesity-associated glomerulopathy [26].

In the present study, we observed that the induction of obesity in C57BL/6 mice caused an increase in BM and an increase in SBP. In agreement with previous studies, the pathophysiology of obesity-associated hypertension depends on multiple factors including diet, metabolism, endothelial and vascular dysfunction, neuroendocrine imbalance, sodium retention, glomerular hyperfiltration, proteinuria, and maladjusted immune and inflammatory responses [27,28]. Obese animals treated with CO showed a decrease in SBP levels and a tendency to reduce body mass.

Evidence is increasing that lipotoxic cell injuries can cause kidney damage, including inflammation, oxidative stress, fibrosis, changes in intracellular signaling pathways, and lipid-induced apoptosis [29]. Our findings reveal that obese mice had altered lipid parameters, altered structural and ultrastructural morphology with loss of renal function, in addition to oxidative stress and increased renal fibrosis and inflammation.

Our findings showed that capybara oil treatment reduced renal fibrosis by decreasing the expression of TGF-β and α-SMA, playing a renal protective role through the suppression of collagen production. Previous studies have indicated that TGF-β acts as one of the key mediators in the progression of pathological renal fibrosis through the production of extracellular matrix, increasing type IV collagen gene expression in tubular epithelial cells, causing renal hypertrophy and fibrosis [30,31,32,33]. One of the fibrotic effects of TGF-β occurs by inducing apoptosis and is associated with podocyte depletion, glomerulosclerosis, loss of glomerular or peritubular capillaries, and tubular atrophy [34,35].

The α-SMA is considered a marker of mature fibroblasts or myofibroblasts. The production of myofibroblasts in the kidney is the critical step that leads to renal interstitial fibrosis [36], where their formation is mediated by the epithelial–mesenchymal transition (EMT). Thus, renal EMT is considered another important pathway in the pathogenesis and progression of renal interstitial fibrosis [37], where there is a loss of epithelial cell adhesion molecules, such as epithelial e-cadherin, which are transformed into mesenchymal marker α-SMA [38,39]. Previous studies have indicated that inhibition of myofibroblast accumulation via EMT is critical in preventing tubulointerstitial fibrosis and preserving renal function [37,38,39]. Studies have shown that the use of omega-3 fatty acids reduced focal tubular atrophy and interstitial fibrosis with suppression of renal α-SMA expression [40,41].

The generation of free radicals is a continuous and physiological process, but excessive production can lead to oxidative damage [42]. In the present study, we observed an increase in the activity of the antioxidant enzymes CAT and SOD in obese animals, indicating an increase in reactive oxygen species (ROS) and, consequently, in oxidative stress. Antioxidant systems exist in the form of enzymatic and non-enzymatic compounds. The enzymatic antioxidant system includes SOD and CAT, for example. These enzymes are responsible for the removal of superoxide anion (O_2_•−) and hydrogen peroxide (H_2_O_2_), respectively [43,44]. The imbalance between the antioxidant and pro-oxidant systems results in oxidative stress, which promotes changes such as lipid peroxidation, DNA fragmentation and oxidation of different molecules, leading to cell death [42]. Under obese conditions, in the presence of excessive ROS production or inflammatory response, the kidney will have a greater chance of being exposed to cellular stress, and may undergo structural changes and loss of function [10,45,46,47].

Regarding the inflammatory profile, we demonstrated a reduction in renal inflammation, with an increase in the expression of IL-10, an anti-inflammatory cytokine, and a decrease in the expression of TNF-α, a pro-inflammatory cytokine, in obese mice treated with capybara oil. TNF-α is an important mediator of inflammation and an important participant in the pathogenesis of kidney injury, promoting apoptosis by inflammation and the accumulation of the extracellular matrix, reducing the glomerular filtration rate and increasing permeability to albumin [48]. IL-10 is a cytokine with important anti-inflammatory properties, such as inhibition of pro-inflammatory cytokine production and stimulation of anti-inflammatory cells, such as regulatory T cells and macrophages [49]. A deficiency in IL-10 expression can worsen the development of CKD through the progression of atherosclerosis [50].

To assess renal function, urinary creatinine and protein levels were analyzed. Creatinine is an important marker of renal function and is mainly derived from muscle creatine metabolism and is excreted entirely in the urine, not being reabsorbed by the body. Therefore, its urinary level decreases when there is a deficiency in renal filtration capacity, suggesting that there is damage to the nephrons [51]. In addition, the presence of protein in the urine, called proteinuria, is toxic to the tubules and can cause tubulointerstitial inflammation and is associated with kidney damage through stimulation of pro-inflammatory effects. Therefore, this biochemical marker is considered a strong parameter to identify the progression of CKD [52]. In our study, the animals in the HFD group had decreased creatinine excretion and proteinuria. Treatment with CO showed a tendency to increase creatinine excretion and decrease proteinuria, but it was not considered a significant difference. Therefore, these data indicate that CO treatment was not able to improve the renal function of obese mice. A previous study corroborates our findings, showing that a diet rich in omega-3 is able to improve kidney injury caused by acute ischemic injury, but is not able to improve kidney function [53].

Urinary excretion of proteins such as albumin causes glomerular diseases such as podocyte damage, mesangial hypertrophy glomerulosclerosis, and vascular endothelial cell dysfunction [54]. The glomerular filtration barrier consists of three layers: the glomerular epithelium, the basal lamina, and the slit diaphragms. Slit diaphragms are formed by the secondary processes of podocytes, the pedicels, and prevent the passage of proteins into the urinary filtrate [55]. Therefore, as a result of podocyte injury and pedicels, the cleft diaphragms rupture and increased urinary protein excretion occurs. Through the transmission and scanning electron microscope, our results indicated that the HFD group presented ultrastructural alterations, such as discontinuity of the fenestrated glomerular capillaries pores and thickening of the glomerular basal lamina, in addition to variable and irregular sizes of the pedicels, thus impairing the functioning of the filtration barrier. Treatment with CO was able to induce a beneficial remodeling of pedicel morphology and improve the integrity of the glomerular basal lamina.

The role of lipid dysregulation in inducing kidney damage in obese individuals has been widely reported [56]. Despite increasing reports associating obesity and high lipid loads with renal failure, the molecular mechanism that governs the development of renal dysfunction, as well as its therapeutic targets, are not fully elucidated. Therefore, there is a need to explore new therapeutic potentials to prevent and/or reverse the harmful effects of obesity and renal pathophysiological changes.

Our study has shown in an unprecedented way that CO improves the pathophysiological kidney damage caused by obesity. Although there are few works in the literature with CO, our group demonstrated that this alternative source of PUFAs was able to reverse the liver damage associated with non-alcoholic fatty liver disease [21] and improve the healing process of wounds in a Swiss mouse model [20]. Several studies already published have observed beneficial effects of supplementation of animal-derived oils in obesity models, especially those rich in essential PUFAs, such as fish oil, mainly due to their prominent anti-obesogenic effects [57,58].

Studies with fish oil relate its beneficial effects in animals fed an HFD [58,59,60]. The mechanisms by which PUFAs are beneficial for obesity and consequent injuries are not well understood, but studies indicate that these fatty acids have effects on the regulation of kinase function proteins and precursors of prostaglandins, leukotrienes and thromboxanes [61].

In previous studies, fish oil supplementation in spontaneously hypertensive rats decreased blood pressure and area of renal injury, in addition to reducing cholesterol and triacylglycerol concentrations in these individuals. The improvement in the lipid pattern through this supplementation is one of the cardiac and renal protective factors found in hypertensive patients [62,63]. Additionally, Kasiske et al. (1991) [64] demonstrated that fish oil supplementation reduced albuminuria, mesangial expansion and glomerulosclerosis in diabetic rats, in addition to decreased serum triacylglycerols and cholesterol and increased HDL. Thus, supplementation with omega-3 was able to alleviate or improve the progressive picture of kidney injury.

However, commercial production of omega-3-rich oils has primarily relied on marine fish oils, which may be hampered by seasonal variations and marine pollution, where growing demand is no longer sustainable [57]. In the current scenario, studies with capybara oil demonstrate a promising alternative to extract the oil in larger quantities and use capybara by-products, since the consumption of its meat is growing every day in Brazil, in addition to being rich in PUFAs [20].

Our model demonstrated that HFD promotes renal lipotoxicity by finding lipid droplets inside renal cells, in association with ultrastructural changes that lead to podocytes, proteinuria and renal dysfunction. The effects of lipotoxicity caused by lipid accumulation were also evidenced in this model, which includes oxidative stress with increased antioxidant enzymes, inflammation and fibrosis of the renal cortex. However, the treatment with CO showed beneficial effects, probably due to its composition and mainly to the high content of omega 3, as already described by Fukushima et al. (1997) [18] and Pinheiro et al. (2001) [19]. Despite not showing improvement in renal function after 4 weeks, CO was efficient in reducing lipid droplets in renal cells and restoring podocytes and pedicels, with restructuring of glomerular filtration. In addition, the treatment exerted antilipotoxic effects such as neutralizing oxidative stress, increasing IL-10 expression, playing an important role in modulating the activity of infiltrating immune cells, and decreasing TNF-α, which led to the reversal of renal fibrosis. Thus, CO presents itself as a potential therapeutic target for obesity-related kidney diseases, but further studies are needed to investigate the composition of this source used and signaling pathways, limiting points of our work.

In conclusion, this study showed for the first time that daily treatment with CO orally promoted a reduction in systemic blood pressure, reestablished renal parenchyma and lipid parameters, decreasing oxidative stress and the expression of pro-inflammatory cytokines in the renal cortex of obese mice with renal disease. In addition, it was able to restore α-SMA and TGF-β levels, with a consequent decrease in renal fibrosis. Therefore, our findings suggest that CO treatment is not only a therapeutic alternative capable of reducing blood pressure, but also has beneficial effects on the structure and ultrastructure of the kidney, improving the systemic and local effects caused by obesity.

## Figures and Tables

**Table 1:** Composition and energy content of the diets (AIN-93M-based diets).

Ingredients (g/Kg)	Control Diet	High-Fat Diet
Casein	140	190
Corn starch	620.69	250.68
Sucrose	100	100
Lard	0	320
Soy oil	40	40
Fiber	50	50
Vitamin mix	10	10
Mineral mix	35	35
Cysteine	1.8	1.8
Choline	2.5	2.5
Antioxidants	0.008	0.016
Total mass	1000	1000
Carbohydrates (% energy)	76	26
Protein (% energy)	14	14
Lipids (% energy)	10	60
Total energy (kcal/kg)	3573	5404

**Table 2:** Biochemical parameters in plasma.

Parameters (mg/dL)	Groups			
	CD	CD + CO	HFD	HFD + CO
Total cholesterol	117 ± 19.5	133 ± 8.6	187.8 ± 15.5 ^a,b,c^	150.5 ± 23.9
HDL	75 ± 5.2	94.2 ± 4.3	76.8 ± 5.7 ^c^	98 ± 15.9 ^a^
LDL	36 ± 9.8	34.6 ± 7.9	80.9 ± 9 ^a,b,c^	63.1 ± 10.6
VLDL	13.6 ± 3	14 ± 2.2	23.3 ± 3.8 ^a,b,c^	15.8 ± 3.2
Triglycerides	62 ± 9.7	64 ± 10.5	107 ± 16.2 ^a,b^	81.5 ± 16.2

Total cholesterol; high density lipoprotein (HDL); low density lipoprotein (LDL); very low density lipoprotein (VLDL) and triglycerides. The HFD group showed increased plasma levels of total cholesterol, LDL, VLDL and triglycerides when compared to the CD and CD + CO groups. The HFD + CO group showed a significant decrease in total cholesterol, LDL and VLDL levels compared to the HFD group. The HFD group showed a significant increase in HDL levels compared to the HFD group and a non-significant decrease in triglyceride levels. (a–c) represent *p* < 0.05 in relation to the CD, CD + CO and HFD groups, respectively. *n* = 6 for all experimental groups.
